# Identifying neuropathologic disease in primary progressive aphasia using narrative speech

**DOI:** 10.1002/alz.71294

**Published:** 2026-03-20

**Authors:** Daniel B. Gutstein, Michael Iorga, Jane Stocks, Nathan Gill, Jennifer Sleeman, Tamar D. Gefen, Michelle Los, Caroline Nelson, Changiz Geula, Sandra Weintraub, Todd Parrish, M. Marsel Mesulam, Elena Barbieri

**Affiliations:** ^1^ Mesulam Institute for Cognitive Neurology and Alzheimer's Disease, Feinberg School of Medicine Northwestern University Chicago Illinois USA; ^2^ Department of Radiology, Feinberg School of Medicine Northwestern University Chicago Illinois USA; ^3^ Department of Psychiatry and Behavioral Sciences, Feinberg School of Medicine Northwestern University Chicago Illinois USA; ^4^ Department of Preventive Medicine Northwestern University Chicago Illinois USA; ^5^ School of Professional Studies Northwestern University Chicago Illinois USA; ^6^ Department of Cell and Developmental Biology, Feinberg School of Medicine Northwestern University Chicago Illinois USA; ^7^ Department of Neurology, Feinberg School of Medicine Northwestern University Chicago Illinois USA; ^8^ Department of Biomedical Engineering Northwestern University Evanston Illinois USA; ^9^ Department of Physical Medicine and Rehabilitation, Feinberg School of Medicine Northwestern University Chicago Illinois USA

**Keywords:** Alzheimer's disease, artificial intelligence, frontotemporal lobar degeneration, narrative speech, neuropathology, primary progressive aphasia

## Abstract

**INTRODUCTION:**

We present an application of artificial intelligence to narrative speech with the primary objective of predicting neuropathologic disease underlying primary progressive aphasia (PPA).

**METHODS:**

Using natural language processing toolkits, features were extracted from transcribed narratives of the Cinderella story. Machine learning ensemble models classified participants as either normal controls (NC) or as individuals with PPA and a subsequent autopsy‐confirmed neuropathologic diagnosis of either Alzheimer's disease (AD) or 4‐repeat tauopathy under the umbrella of frontotemporal lobar degeneration (FTLD‐4Rtau).

**RESULTS:**

All models successfully distinguished transcribed narratives of AD from those with FTLD‐4Rtau, as well as the narratives of NC from those with PPA. Feature permutation revealed diverging patterns of contribution to classification depending upon language domain and disease pathology.

**DISCUSSION:**

The usage of artificial intelligence in the context of naturalistic language tasks may ultimately serve as a complementary aid in differential diagnosis of PPA disease pathologies and in uncovering avenues for disease‐specific interventions.

## Highlights

- We introduce a novel application of natural language processing and machine learning upon narrative speech transcriptions to distinguish both individuals with primary progressive aphasia (PPA) from cognitively healthy, age‐matched participants as well as to differentiate between groups of PPA with underlying disease pathologies of Alzheimer's disease or 4‐repeat tauopathy under the umbrella of frontotemporal lobar degeneration.
- This analysis is the first to apply artificial intelligence (AI) to narrative speech as a mechanism for differentiating confirmed disease pathologies within PPA.
- The effective classification of clinical status and disease pathology within PPA, together with the permutation of natural language features, revealed granular patterns in speech production, thereby demonstrating how predictive modeling can map specific features of narrative speech in PPA.
- Our models connecting narrative speech to both clinical status and underlying disease pathology within PPA highlight the potential utility of AI to aid in the detection, diagnosis, and management of disease‐specific speech impairments.

## 1 INTRODUCTION

Narrative speech is arguably the most representative measure of a patient's natural speaking ability, providing extensive information regarding acquired language disorders such as primary progressive aphasia (PPA), a clinical syndrome due to neurodegenerative disease defined by the gradual deterioration of language functions.^1^, ^2^ Prior research has identified three major clinical variants of PPA: semantic (svPPA), agrammatic (agPPA), and logopenic (lvPPA).^3^ svPPA is characterized by profound naming and word comprehension impairment.^3^, ^4^, ^5^, ^6^ In line with previously published guidelines, agPPA is defined by deficits in grammatical components of language, including morphology (e.g., use of prepositions, articles, and verb inflections) and syntax (e.g., ordering of words within a sentence, use of subordination), which may co‐occur with articulatory deficits, mostly in the form of apraxia of speech. lvPPA differs from svPPA and agPPA in that difficulties in word finding occur without significant impairment of word comprehension or morpho‐syntax and may be accompanied by difficulties in repetition.^4^, ^5^, ^6^, ^7^

Measures of narrative production have been used^8^ to provide detailed linguistic profiles of each PPA variant^9^, ^10^, ^11^ and to differentiate among variants.^12^, ^13^, ^14^ These studies indicate *that articulatory precision and morpho‐syntax* are primarily affected in individuals with agPPA,^9^, ^12^, ^15^, ^16^ although impairments may also occur in lvPPA.^11^, ^16^, ^17^ Measures of fluency and phonological errors are typically abnormal in both agPPA and lvPPA.^9^, ^12^, ^15^, ^16^ In contrast, individuals with svPPA experience disruptions in measures of lexical content,^15^, ^17^, ^18^ meaning, for example, that they produce more pronouns and verbs as well as words with higher (vs. lower) frequency.

Several studies have applied natural language processing (NLP) to discriminate among PPA clinical variants^14^, ^19^, ^20^, ^21^, ^22^ by focusing on morphosyntactic features such as subordinating conjunctions, verb inflections, and various forms of pronouns,^14^ while other research has sought to distinguish PPA from other forms of dementia^23^ by analyzing bigrams and measures such as content word frequency. However, recent developments suggest that the association between PPA clinical variant and underlying neuropathology is mostly probabilistic.^24^, ^25^, ^26^, ^27^, ^28^, ^29^ While variants may show a preferred pathological association, this mapping is not straightforward, and the degree of pathological specificity varies substantially across variants. In particular, agPPA exhibits marked neuropathological heterogeneity, limiting the utility of clinical variant labels as proxies for underlying pathology. These observations motivate analytic approaches that focus directly on predicting neuropathology, independent of clinical diagnosis.

**RESEARCH‐IN‐CONTEXT**

1. **Systemic review**: The authors reviewed the literature describing the extent of language‐based impairment in the main forms of primary progressive aphasia (PPA) and discussed the imperative to focus upon disease pathologies. While natural language processing (NLP) and machine learning (ML) tools have been previously applied to understand language impairment in the context of PPA clinical subtypes, no study has used an NLP and ML framework to identify language biomarkers of disease pathology in groups of autopsied individuals with PPA. The relevant references are cited appropriately.
2. **Interpretation**: Model outcomes highlight nuanced patterns in speech production, enabling the differentiation of clinical status as well as that of disease pathology within the PPA group. The results demonstrate the utility of models to both diagnose PPA down to the underlying disease and to uncover overlapping and discriminant speech patterns.
3. **Future directions**: Future work will broaden the scope of disease pathologies as well as neurological conditions and shall aim to include acoustic measures and speech rate data to facilitate the development of more advanced models encapsulating a more comprehensive scope of aphasic inhibition.

While magnetic resonance (MR) imaging can be used to distinguish PPA due to TAR DNA‐binding protein 43 (TDP‐43) Type C pathology because of the almost unique association between left‐lateralized atrophy of the temporal pole and early impairment of single‐word comprehension,^30^, ^31^, ^32^, ^33^ the detection of other disease pathologies is more difficult.^25^ Fluid and positron emission tomography (PET) imaging biomarkers are commonly used to predict Alzheimer's disease (AD) pathology,^34^, ^35^ though some studies have noted that the known relationships between positivity to AD biomarkers and clinical manifestations of PPA are not deterministic.^36^ Additionally, new methods aimed at the in vivo detection of Frontotemporal Lobar Degeneration (FTLD) 3‐repeat and 4‐repeat tauopathies (FTLD‐3Rtau, FTLD‐4Rtau are still in development.^37^, ^38^ Finally, PET scans may not be safe or tolerated for all individuals. These challenges highlight the need to enhance diagnostic toolkits via the development of safe and accurate tools for early prediction of the underlying disease pathology. Accordingly, a recent study^39^ successfully selected PPA patients with an elevated probability of a positive PET amyloid imaging result based solely upon quantitative connected speech features extracted from spontaneous speech samples, thereby pointing to speech and language markers as viable inputs for augmenting current diagnostic methods.

The current study evaluates whether speech features extracted from narrative speech using machine learning (ML) models have the potential to be incorporated within a diagnostic toolbox as safe, in vivo biomarkers of disease pathology. The following sections describe an analysis of narrative speech on individuals with PPA confirmed at autopsy to have one of two underlying disease pathologies—AD or FTLD‐4Rtau—as well as healthy individuals. The approach focuses on the usage of narrative speech samples to differentiate autopsy‐confirmed neuropathologic disease of AD from those with FTLD‐4Rtau within a cohort of PPA patients. Classification of individuals with PPA against cognitively healthy participants was carried out to establish baseline model sensitivity to language impairment. This analysis establishes a proof of concept for a quantitative narrative speech analysis framework designed to facilitate differential diagnosis and characterize disease‐specific language impairments in PPA.

## 2 METHODS

### 2.1 Stratification of participants

**FIGURE 1:**
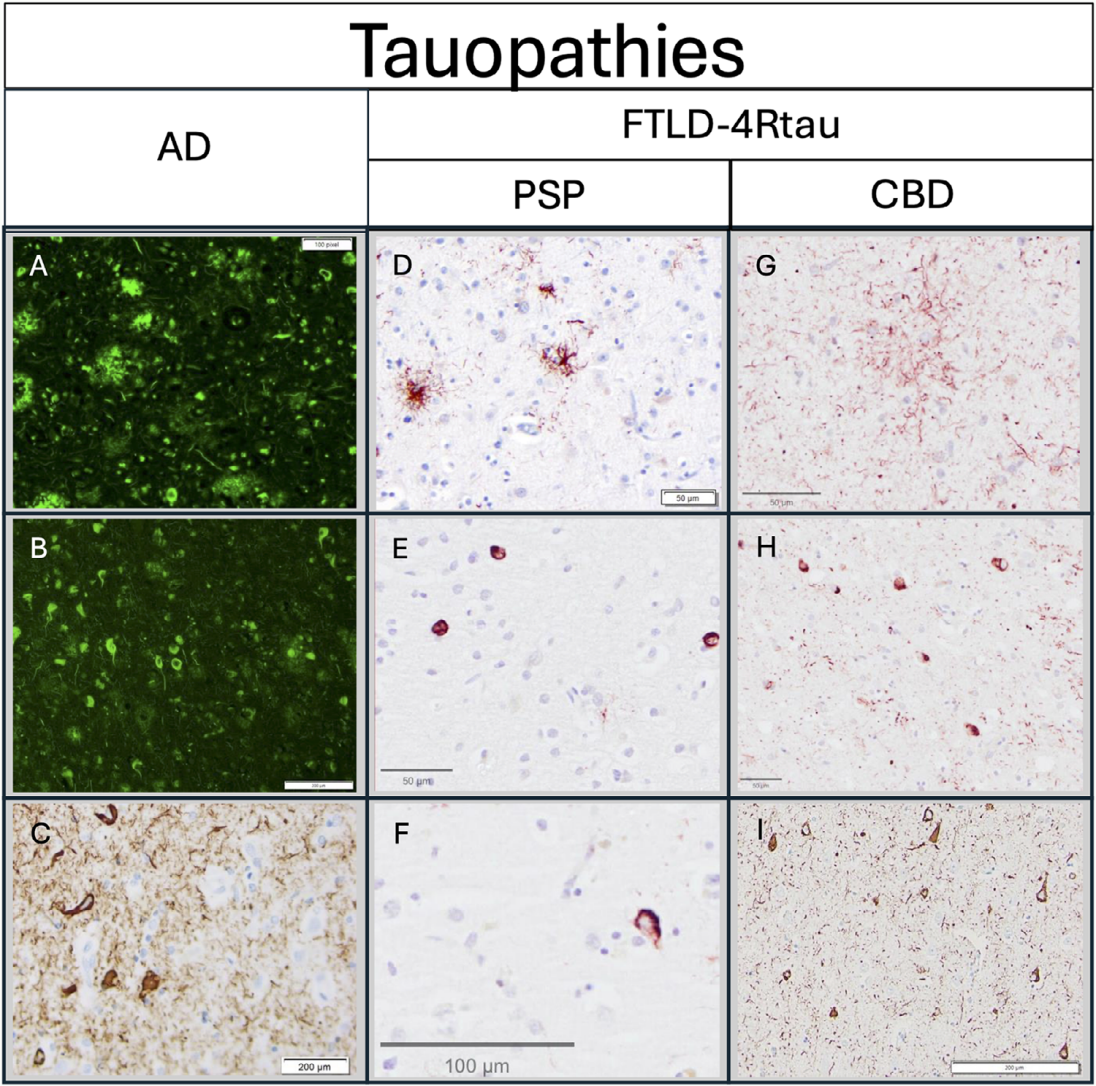
Hallmark pathologic features of AD and FTLD‐4Rtau proteinopathies. Staining of *post mortem* tissue depicts characteristic thioflavin‐S‐positive (A) Aβ plaques and (B) neurofibrillary tau tangles in the middle frontal gyrus of participants with *post mortem* primary neuropathologic diagnosis of AD. Scale bar: A = 100 pixels, B = 200 µm. AT‐8 immunohistochemistry also illustrates neurofibrillary tangles (C) in AD cases in the entorhinal cortex. Scale bar = 200 µm. In FTLD‐4Rtau cases with PSP, AT‐8 staining in the frontal cortex reveals characteristic tufted astrocytes (D), neuronal inclusions (E), and coiled bodies (F). Scale bar: D,E = 50 µm, F = 100 µm. In FTLD‐4Rtau cases with CBD, AT‐8 staining indicates the presence of astrocytic plaques (G), neuronal inclusions, and frequent neuropil threads (H), in the middle frontal gyrus. Scale bar: G,H = 50 µm. In the entorhinal cortex, AT‐8 staining reveals neuronal inclusions and frequent neuropil threads (I). Scalebar = 200 µm. Aβ, amyloid beta; AD, Alzheimer's disease; CBD, corticobasal degeneration; FTLD‐4Rtau, frontotemporal lobar degeneration with 4‐repeat tauopathy; PSP, progressive supranuclear palsy.

The data reported in this study were collected from participants enrolled in the longitudinal Northwestern University PPA Research Program. Fifteen cognitively healthy, living age‐matched normal controls (NC) were included, as well as 77 individuals diagnosed with PPA with either an autopsy‐confirmed (*n* = 54) or clinically inferred (*n* = 23) pathological diagnosis of AD or FTLD‐4Rtau, as described below. Cognitively healthy participants were recruited from the greater Chicago area and screened over the phone to determine MR imaging (MRI) safety and cognitive status. For the latter, a short (≈ 30 minutes) neuropsychological test battery (used in other studies and validated at the Mesulam Institute^40^) was administered in accordance with institutional review board protocol after the provision of verbal consent. Clinical diagnosis of PPA was determined by a behavioral neurologist, based on the presence of a clinical history of progressive language impairment that spared other cognitive functions for the first 1 to 2 years after symptom onset.^1^, ^2^ The neuropathologic findings were available for 54 individuals (*N*_AD _= 37, *N*_FTLD‐4Rtau _= 17) with clinically diagnosed PPA who had previously committed to brain donation. After autopsy, the cerebral hemispheres were separated in the midsagittal plane, cut into 2 to 3 cm coronal slabs, fixed in formalin for 2 weeks or 4% paraformaldehyde for 36 hours, taken through sucrose gradients (10%–40%) for cryoprotection, and stored in 40% sucrose with 0.02% sodium azide at 4°C. Semiquantitative neuropathological evaluations of gross atrophy, pathologic inclusions, neuronal loss and gliosis, and superficial microvacuolation were performed at autopsy. Neuropathological diagnoses of FTLD‐tau subtypes were rendered according to Cairns et al.^41^ or published criteria for AD.^42^, ^43^ Hallmark pathologic features of AD and FTLD‐4Rtau tauopathies, derived from frontal and entorhinal cortices, are outlined in Figure 1.

Data from 23 additional patients (*N*_AD _= 17, *N*_FTLD‐4Rtau _= 6) with a clinical diagnosis of PPA but without autopsy‐confirmed neuropathologic diagnoses were included for the purpose of augmenting the training set. These inferred pathological diagnoses were excluded from the validation data and therefore were not used to evaluate our models. The underlying neuropathology for each subject within this additional subset was inferred by trained experts (M.M.M. and E.B.) based on a comprehensive evaluation of imaging and/or fluid biomarkers, that is, atrophy and hypometabolism patterns as indexed by structural MRI and fluorodeoxyglucose PET, respectively, amyloid PET/tau PET, and cerebrospinal fluid (CSF) analysis. Specifically, AD pathology was inferred when there was substantial cortical temporo‐parietal atrophy or hypometabolism in conjunction with either a clinical diagnosis of lvPPA or presence of fluid or imaging biomarkers consistent with AD. Conversely, the presence of FTLD‐4Rtau pathology was inferred from the absence of significant cortical atrophy at the first visit, in conjunction with a clinical diagnosis of agPPA and negative evidence of AD on fluid/imaging biomarkers. AD positivity on fluid/imaging biomarkers was determined either through the review of medical records (for CSF or clinically ordered amyloid PET) or based upon clinical reads provided by at least two clinically certified radiologists (for amyloid PET or tau PET scans acquired as part of the research protocols at the Mesulam Institute).

**FIGURE 2:**
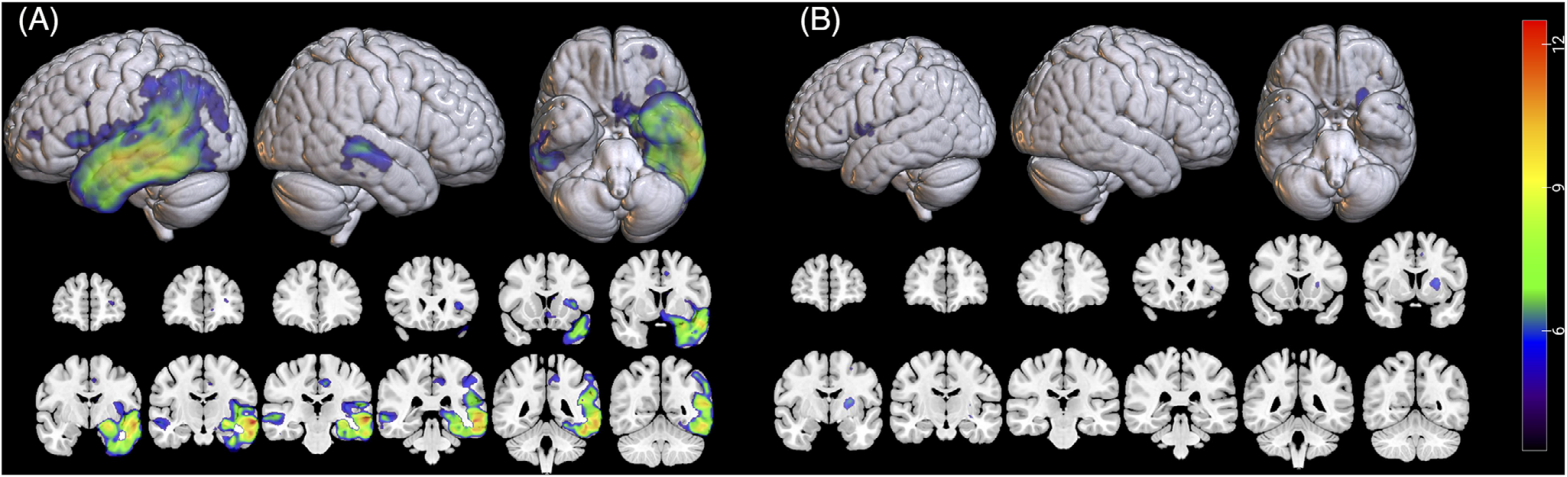
Regions of significant gray matter atrophy. Atrophy maps for the two PPA groups with (A) confirmed AD pathology (*n* = 37) or (B) confirmed FTLD‐4Rtau pathology (*n* = 17) relative to a group of 82 cognitively healthy individuals. Atrophy maps were calculated using two‐sample *t* tests, based on the first available magnetic resonance imaging scan of each PPA participant. A statistical threshold of *P* < 0.05, corrected for multiple comparisons using family‐wise error, was applied at the voxel level, in addition to a cluster size threshold of *k* > 10. FTLD‐4Rtau, frontotemporal lobar degeneration with 4‐repeat tauopathy; PPA, primary progressive aphasia.

Figure 2 illustrates regions of significant gray matter atrophy in the two groups with confirmed disease pathology, relative to a cohort of 82 middle‐aged cognitively healthy controls. Atrophy maps were obtained from high‐resolution 3T magnetization‐prepared rapid gradient echo scans performed on each participant's first visit and therefore display *ante mortem* atrophy patterns in the early stages of the disease. In individuals with confirmed AD pathology, left‐hemisphere cortical atrophy affected the entire left temporal lobe, including parts of the insula, inferior frontal gyrus (IFG), left angular and posterior supramarginal, left posterior cingulate gyrus and precuneus, as well as the entorhinal and perirhinal cortices and the parahippocampal and fusiform gyri. Right hemisphere atrophy was mostly limited to the mid‐posterior superior temporal gyrus. In participants with confirmed FTLD‐4Rtau, peak atrophy occurred in the left putamen. There were additional minimal areas of cortical atrophy within the left IFG, the frontal operculum, and the left middle frontal gyrus. Medially, some atrophy was present in the left anterior cingulate.

Group differences in study variables were evaluated using chi‐squared tests for categorical variables and analysis of variance for continuous variables. Where appropriate, post hoc pairwise comparisons were adjusted for multiple testing using the Holm correction. Relevant demographic data pertaining to disease pathology, clinical status, age, sex, education, and race are presented in Table 1. Briefly, FTLD‐4Rtau and AD groups did not significantly differ in age at disease onset (*P *= 0.2), clinical severity (Clinical Dementia Rating, *P *= 0.3), or aphasia severity (Western Aphasia Battery aphasia quotient, *P *= 0.2). All individuals with PPA included in the study, both confirmed and inferred, were deemed to be either AD or FTLD‐4Rtau; none had evidence of mixed pathologies. Though inferred designations of FTLD‐4Rtau, as noted, were made in part due to the absence of in vivo biomarkers whose presence (such as that of amyloid plaques) would imply the presence of AD pathology, the absence of comorbid disease pathologies cannot be confirmed until the time of autopsy. PPA groups did not differ from the NC group with respect to education, race, ethnicity, or sex, though the PPA groups were slightly older than the NC group.

**TABLE 1:** Subtype, age, and language scores for individuals with PPA.

	Clinical subtype			Age		Sex (%F)	CDR	WAB‐AQ	Edu	Race (%White)
	agPPA	lvPPA	Mixed^a^/Other^b^	At visit	At onset					
AD	15	14	8	66.1 (6.8)	61.4 (7.3)	38	0.3 (0.2)	84.5 (8.7)	16.4 (2.3)	100
AD‐inf	4	11	2	67.0 (5.3)	62.8 (5.0)	29	0.2 (0.3)	88.1 (7.6)	16.3 (2.3)	94
FTLD‐4Rtau	14	2	1	68.1 (8.0)	64.7 (8.6)	47	0.2 (0.3)	85.0 (7.3)	15.4 (2.1)	100
FTLD‐4Rtau‐inf	6	0	0	70.2 (4.3)	67.2 (3.8)	66	0.1 (0.2)	85.4 (1.6)	14.7 (2.1)	100
NC	–	–	–	61.8 (6.0)	–	40	0.0 (0.0)	–	15.8 (2.3)	80

*Note*: Results presented as mean (standard deviation) unless otherwise noted.

^a^“Mixed” diagnosis is defined by Mesulam et al. (2009) criteria, where patient displays deficits in both grammar and verbal semantics.

^b^“Other” includes patients who were either too mild to fully characterize at an initial visit or who presented with features of both agrammatic and logopenic PPA.

Abbreviations: AD, Alzheimer's disease; agPPA, agrammatic primary progressive aphasia; CDR, Clinical Dementia Rating Scale (Global); FTLD‐4Rtau, 4‐repeat tauopathy under the umbrella of frontotemporal lobar degeneration; inf, inferred pathology; lvPPA, logopenic primary progressive aphasia; M/O, mixed/other; NC, cognitively healthy normal control; PPA, primary progressive aphasia; WAB‐AQ, Western Aphasia Battery aphasia quotient; Edu, education.

### 2.2 Collection of narrative speech samples

Participants were shown a wordless flipbook depicting the Cinderella story and asked to inspect the pages in silence to remind themselves of the narrative. After the viewing period (so long as each participant desired), the examiner would close the book and prompt the participant to retell the story from memory. Cues were provided whenever needed to remind participants of the task or to redirect the narrative in cases in which participants would fixate on irrelevant details or stray to other topics. All speech samples were audio recorded and manually transcribed by trained personnel in the Aphasia and Neurolinguistics Research Laboratory, following procedures described elsewhere.^16^, ^44^ Preprocessing of the transcriptions included anonymization and removal of speech belonging to the task proctor.

### 2.3 Feature extraction with NLP

**FIGURE 3:**
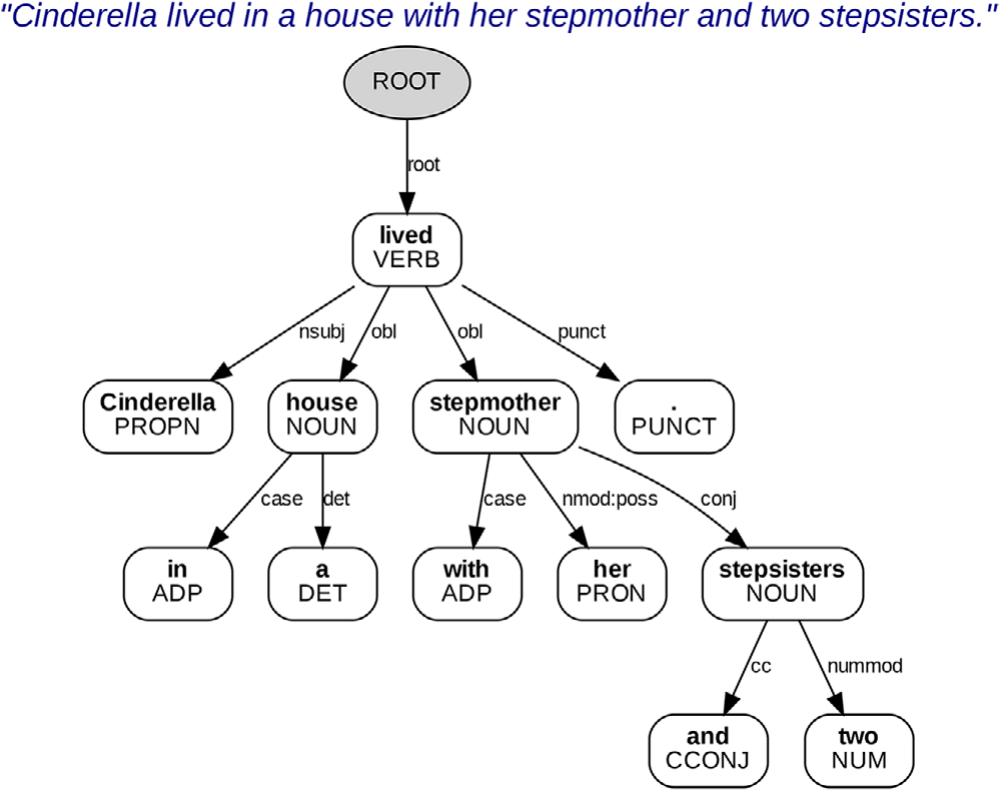
Sample dependency tree based upon a detailed characterization of dependency features describing grammatical relationships extracted using Stanza.

NLP packages were used to extract structured features from transcriptions of the narrative speech samples. Features were split into two separate datasets, one consisting of classical linguistic features (e.g., part of speech, dependencies) and the other of transformer embeddings, that is, numerical vector representations of text that simultaneously capture multiple aspects of language (including, e.g., meaning and syntax). Publicly available NLP toolkits—SpaCy^45^ (version 3.8.7) in conjunction with the TextDescriptives^46^ (version 2.8.4) package, as well as Stanza^47^ (version 1.10.1)—were leveraged to extract a broad set of text features from the Cinderella story transcriptions. SpaCy and the TextDescriptives package generated labeled features encompassing classical linguistic metrics (e.g., number of nouns, verbs, etc.), which offered a coarse evaluation of the overall sentence structure. Stanza contributed labeled dependency features characterizing the grammatical relationships between words in a sentence (see Figure 3 for an example). This information is particularly relevant in describing grammatical errors, which we hypothesized were important to the classification tasks.

Transformer embeddings from Bidirectional Encoder Representations from Transformers (BERT base‐uncased)^48^—a large language model that pretrains deep bidirectional representations from unlabeled text and can perform language inference—encoded additional high‐order linguistic information. Transformer embeddings have the advantages of being context dependent and of incorporating a wide range of linguistic and semantic properties, including phrase‐level dependencies, implicit meaning, syntactic relationships, discourse coherence, and sentence structure. BERT was used to encode each individual participant's narrative speech sample, and the extracted numeric vectors from BERT were then concatenated into a single multidimensional embeddings matrix.

In each of the two feature datasets, we aimed to capture global discourse properties, and syntactic and lexical patterns, allowing for a richly nuanced evaluation of speech features potentially altered in PPA. To facilitate the subsequent interpretability analysis, which would have been skewed by latent multicollinearity between the high‐dimensional numeric transformer embeddings and the more classical linguistic features derived from SpaCy and Stanza, the dataset was split in two—separating the classical linguistic features from the transformer embeddings.

### 2.4 Structure of classification models

Machine learning models were designed to perform three‐way classification of narrative speech samples belonging to either the FTLD‐4Rtau, the AD, or the NC group. The classification models were defined as large ensembles of small neural networks, each comprising a single hidden layer with three neurons. This choice was motivated by preliminary testing showing that increases in the number of neurons or the number of hidden layers led to overfitting and reduced performance. Final model predictions were made by taking the mode across 100 independently trained networks, a number chosen based on the evidence of negligible performance improvement when the ensemble was expanded beyond 100 models. This basic architecture was chosen given the limited dataset size (*N*_predicted + confirmed_ = 92).

The networks were implemented using the MLPClassifier library from the scikit‐learn Python package^49^ with a rectified linear unit (ReLU) activation function. Training was conducted using the Adam optimizer, with an initial learning rate of 0.001 and without microbatching (batch size was the entire training dataset).

**FIGURE 4:**
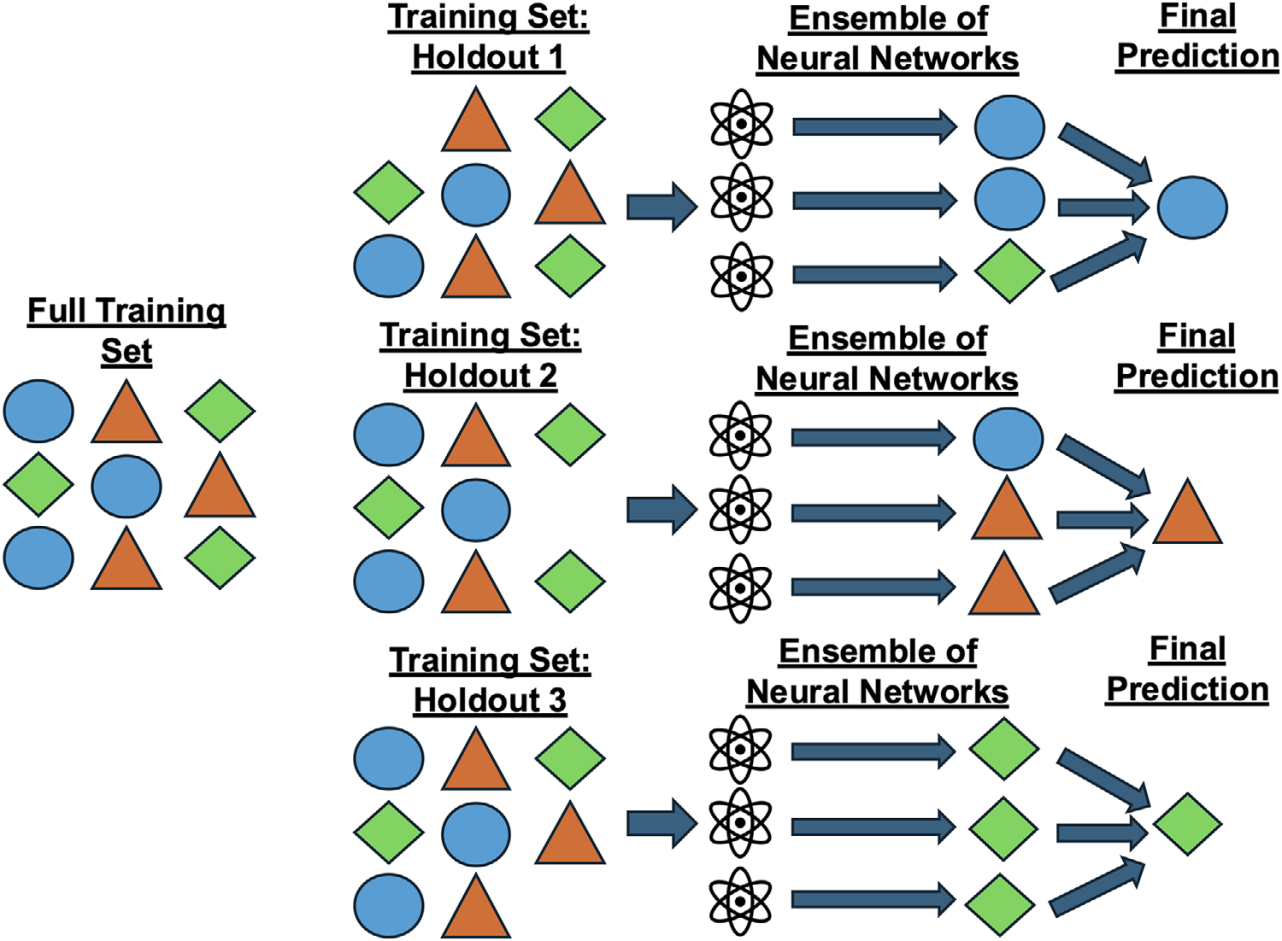
Design of neural network ensemble. The training set is broken down into *n* sets, corresponding to the number of participants who are cognitively healthy or who have primary progressive aphasia (PPA) and confirmed disease pathology based upon autopsy. Each training set also includes the data from the 23 living individuals with PPA and the corresponding inferred disease pathologies. Within each training group, 100 neural networks independently learn the connections between linguistic inputs and the disease class. Each trained ensemble then makes a prediction of disease class for the single hold‐out participant. The final prediction is based upon the “mode” prediction of disease class for each hold‐out.

To assess the generalizability of the trained ensembles, we used a leave‐one‐out cross‐validation approach. Neural networks were initialized with randomly sampled seeds and trained on non‐stratified, scaled data to perform multiclass prediction and classify participants into three groups: individuals with PPA and autopsy‐confirmed AD or FTLD‐4Rtau, or NC. Although the focus of the study was on the differentiation of AD from FTLD‐4Rtau within the PPA cohort, the differentiation of NC from the two PPA groups was considered a necessary step to establish the clinical validity of the method, which must be accurate in differentiating the speech of cognitively healthy individuals from that of individuals diagnosed with PPA. The 23 additional participants with predicted neuropathologic disease were included in the training set for every iteration but were excluded from validation, as their labels could not be independently confirmed. Thus, as previously established, the methodological design optimized predictive power by augmenting the dataset without diluting the integrity of the validation dataset by using probabilistic labels. Figure 4 illustrates a conceptual breakdown of the neural network ensemble design, whose simple structure—which did not use class weighting or resampling—combined with majority‐based voting was set up to facilitate statistically meaningful and balanced results.

### 2.5 Evaluation of model performance

The separate analyses performed on each of the two feature sets—classical linguistic measures and transformer embeddings—were evaluated both against chance and one another. Model performance for every class was assessed using the F1‐score (f‐score), a composite metric that balances precision and recall and is robust to class imbalance. While the inclusion of 92 participants (training + inferred) is small for a three‐way classification task, the power of our findings, further elucidated in the Results and Discussion sections, assessed whether there was a sufficient effect size to counterbalance the limitations inherent in using a small dataset. Accordingly, Fisher exact tests were applied to each model, using a one versus all approach for each class, to determine whether performance exceeded chance levels. To directly compare the respective predictive capacities of the two models, we used exact McNemar‐type tests^50^ (α = 0.05, one‐sided) within a one versus all framework, to assess whether the transformer model significantly outperformed the classical model.

### 2.6 Manual categorization of classical features

To facilitate interpretability and explanatory analysis of model performance, the 93 classical linguistic features from SpaCy and Stanza were categorized into 13 thematic subclusters. Subclusters were manually defined—exclusively based on theoretical knowledge—by an expert in psycholinguistics (E.B.) into the following categories: (1) fluency and output amount; (2) coherence—defined as the similarity between sentences in close proximity to one another; (3) lexical diversity, heterogeneity, and similarity between words used in the text; (4) features describing open‐class words (nouns, verbs, adjectives, and adverbs), which are word classes that are typically richer in meaning; (5) features describing closed‐class words (prepositions, articles, pronouns, etc.), which are word classes that are typically less rich in meaning but serve grammatical functions; (6) features describing other parts of speech; (7) dependency features involving relationships within noun phrases; (8) dependency features involving relationships within verb phrases; (9) dependency features involving relationships within conjunctions/prepositional phrases; (10) dependency features involving argument structure relationships; (11) features describing syntactic dependencies between clauses; (12) features describing dependency distance, which pertains to the distance between a dependent and its head; and (13) features describing other dependencies where relationships between words could not be clearly categorized. Transformer variables were excluded from the clustering procedure because these features are not inherently interpretable without the aid of advanced methods.

### 2.7 Estimation of individual variable importance

To evaluate the contribution of each of the classical linguistic variables to classification accuracy, individual features were permuted (one at a time) from the dataset and the relative drop in model performance was used as a measure of each feature's impact on the f‐score of the overall model. We implemented a permutation‐based method in which each variable was independently shuffled across the entire dataset while all other inputs remained static. Variable importance was quantified by the decrease in model performance (as measured by f‐score) relative to the full classical model. Variables with greater influence on model predictions yielded larger drops in performance when permuted. This permutation process was repeated 30 times for each variable. To ensure the robustness of estimates, the resulting f‐scores were resampled with replacement, and 95% confidence intervals were derived via 1000 bootstrap iterations of the permuted f‐scores. A variable's contribution was considered significant—unadjusted for multiple comparisons—if the upper bound of its confidence interval fell at or below the performance of the full model. Our innovative permutation‐based approach overcomes the challenge of assessing individual variable importance in neural networks, where features and the ReLU activation function display non‐linear interactions.

## 3 RESULTS

### 3.1 Comparison of statistical models

**FIGURE 5:**
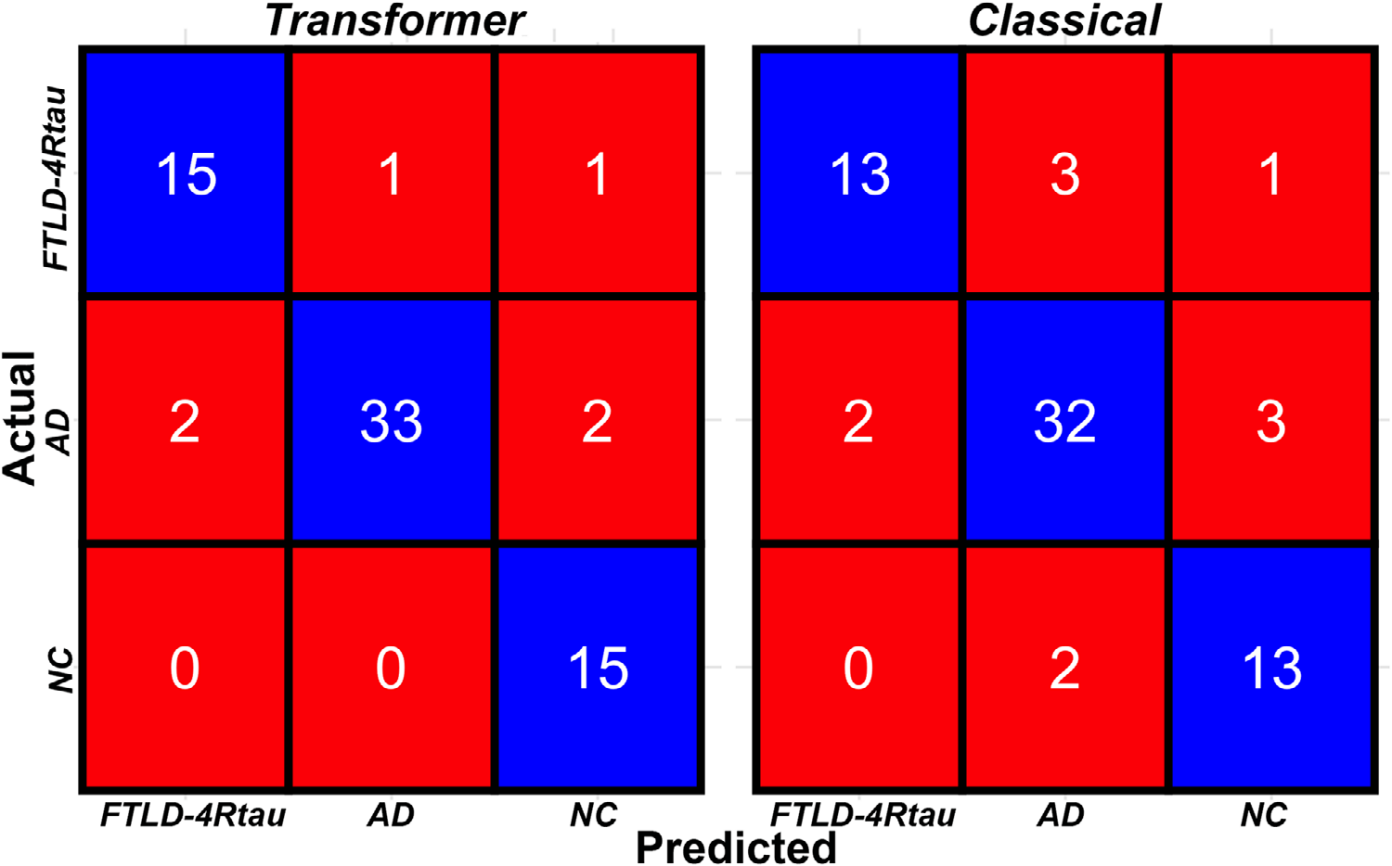
Confusion matrices representing the final validation output from the transformer and classical models. Accurate predictions for each model are indicated along the blue diagonal in each matrix. AD, Alzheimer's disease; FTLD‐4Rtau, 4‐repeat tauopathy under the umbrella of frontotemporal lobar degeneration; NC, normal control.

The results of the two models—(1) transformer and (2) classical features—are presented in Table 2. Both models demonstrated statistically significant differentiation among the three predicted classes. The optimized transformer model achieved f‐scores of 88% (75%, 98%) for FTLD‐4Rtau, 93% for AD (87%, 99%), and 91% (78%, 100%) for NC. Both models performed significantly better than chance (Fisher exact test, *P* < 0.05) in differentiating cognitively healthy individuals from individuals with PPA and in distinguishing among neuropathology‐based PPA groups. The retrained model using only classical linguistic features (dependencies, coherence, counts of word types, lexical diversity, and parts of speech) yielded slightly lower f‐scores of 81% for FTLD‐4Rtau (63%, 94%), 87% (77%, 94%) for AD, and 81% (64%, 94%) for NC, though no significant difference was found between the two models in either overall performance or within individual classes (exact McNemar type test, *P* > 0.05). Thus, while the transformer model produced superior results across classes, there was insufficient evidence to conclude that the transformer model was significantly better than the classical model. Confusion matrices of the final validation output from the transformer and classical models are presented in Figure 5. Both models demonstrated well‐balanced classification in terms of precision and recall across the three groups of interest, suggesting that the issue of class imbalance was successfully mitigated.

**TABLE 2:** Validation f‐scores (and 95% confidence intervals) of the two trained models across the three classes.

	FTLD‐4Rtau	AD	NC
Transformer model	88% (75%, 98%)	93% (87%, 99%)	91% (78%, 100%)
Classical model	81% (63%, 94%)	87% (77%, 94%)	81% (64%, 94%)

Abbreviations: AD, Alzheimer's disease; FTLD‐4Rtau, 4‐repeat tauopathy under the umbrella of frontotemporal lobar degeneration; NC, cognitively healthy normal control.

### 3.2 Individual variable importance as observed following feature permutation

**FIGURE 6:**
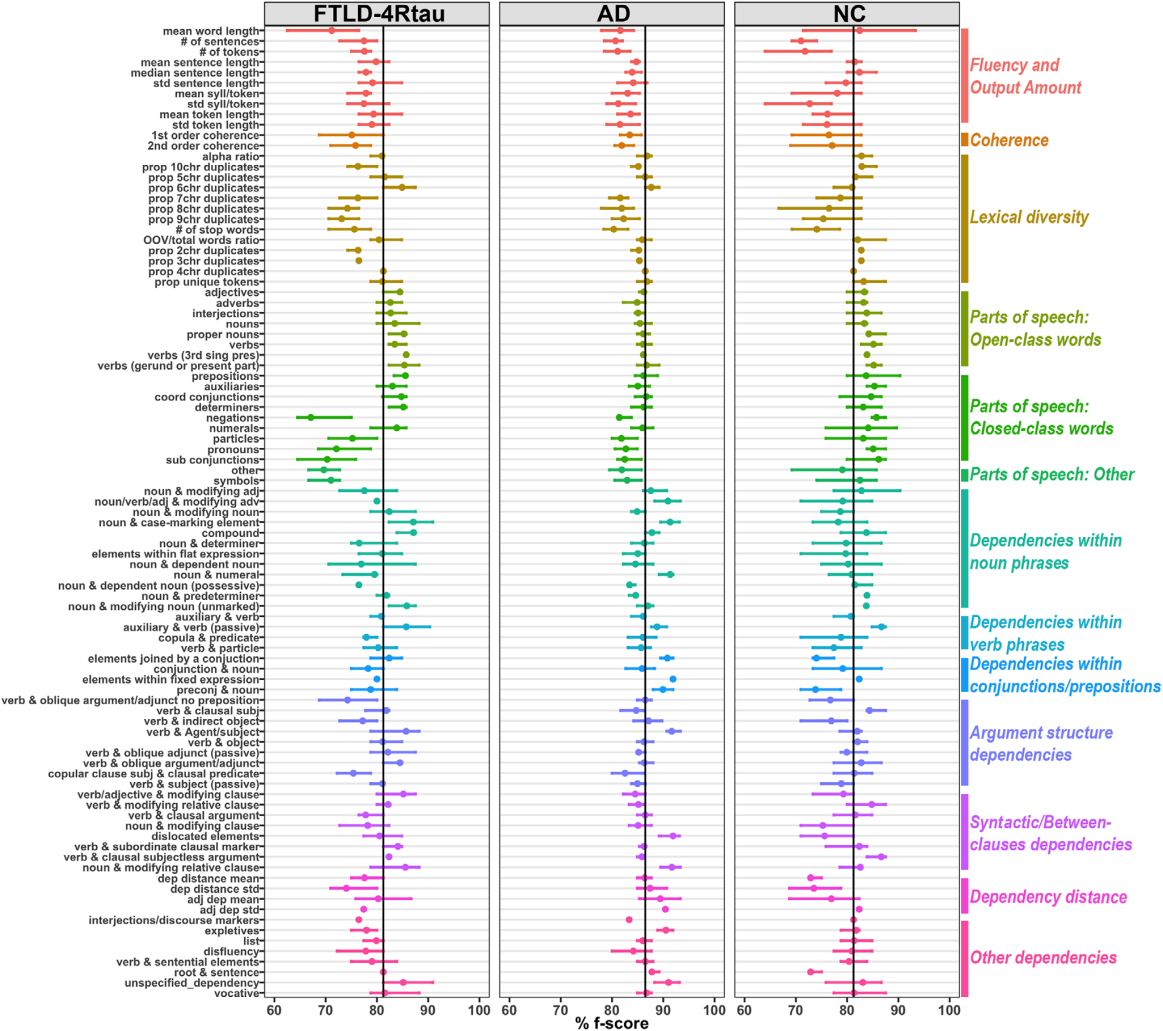
Feature permutation analysis. Mean percentage f‐scores (round points) and 95% confidence intervals (bars) in predicting each variable, for pretrained classical model when each variable was permuted/sampled without replacement. Colors mark subgroups of variables. Black vertical lines mark the overall f‐score of the full classical model before the introduction of any post hoc individual variable permutation. AD, Alzheimer's disease; adj, adjective; adj dep,  adjacent dependency; adv,  adverb; chr,  character; coord,  coordinating; dep, dependency; FTLD‐4Rtau, frontotemporal lobar degeneration with 4‐repeat tauopathy; NC, normal control; OOV, out‐of‐vocabulary; preconj, element preceding a conjunction; pres, present; sing, singular; subj, subject; syll, syllables

The results of the permutation approach are illustrated in Figure 6 and are also detailed in the supporting information (Table S1) along with a full list of classical variables, variable descriptions, and variable cluster assignments. Among feature categories, dependency information emerged as the most influential feature group in the classical model, as permutation tests revealed the largest relative performance drop. F‐scores within the FTLD‐4Rtau group were primarily influenced by the proportion of out‐of‐vocabulary words (suggestive of paraphasias), as well as the use of negations (“not,” “none”) and subordinating conjunctions (e.g., “after,” “since”). Production of other closed class words such as pronouns (e.g., her, he) and particles (e.g., possessive markers, “not”) also contributed, though to a lesser extent. Among dependencies, those most predictive of classification accuracy for this group included: (1) argument structure dependencies describing the relationship between verbs and their objects or adjuncts, or between a grammatical subject and a clausal predicate (e.g., “That Cinderella danced with the prince surprised her stepsisters”); (2) dependencies marking the relationship between a noun and a genitive complement within a possessive (e.g., “Cinderella’s slipper was lost”); and (3) overall dependency distance. Other variables associated with the prediction of FTLD‐4Rtau—though weaker—included features pertaining to the fluency and output amount and lexical diversity, as well as those reflecting the presence of fillers (e.g., “you know”) and coherence (i.e., similarity in content/structure between consecutive sentences).

F‐scores within the AD group were primarily driven by features pertaining to the amount of speech output and lexical diversity, along with the use of negations and closed class words such as pronouns and particles. In contrast to the FTLD‐4Rtau group, dependency‐based features made only a marginal contribution to the classification of the AD group. The most significant features among dependencies were those marking the relationship between a noun and a genitive complement within a possessive and those linking a noun to its determiner (e.g., The evil stepsisters). Notably, average dependency distance and verb–argument structure—which were predictive of FTLD‐4Rtau and NC—did not significantly influence the classification of AD.

F‐scores within the cognitively healthy control group were primarily driven by a limited number of linguistic features, most of which pertained to fluency and output amount (e.g., total number of tokens, total number of sentences) or dependencies. Among dependency features, those referring to the relation between two elements of a conjunction (e.g., princes and princesses) or between the head of the coordination and the first element in a conjunction (e.g., both the stepsisters and Cinderella) were most predictive, together with the average dependency distance and the dependency marking the relation between the verb and its indirect object (e.g., The fairy godmother gave Cinderella a carriage and a coachman).

## 4 DISCUSSION

This study uses narrative speech samples as inputs to differentiate PPA due to AD pathology from PPA due to FTLD‐4Rtau pathology, as well as PPA from cognitively healthy participants. While previous studies have either distinguished PPA from other dementias,^23^ discriminated among PPA subtypes and/or between PPA and cognitively healthy individuals,^14^, ^19^, ^20^, ^21^, ^22^ or predicted amyloid positivity on amyloid PET imaging,^39^ this is the first study leveraging narrative speech to predict autopsy‐confirmed disease pathologies within the context of a single clinical syndrome. Accordingly, both the high‐dimensional transformer space—which captures word meaning, syntactic relationships, discourse coherence, and sentence structure—and the classical linguistics feature space successfully differentiated disease pathologies within PPA while also distinguishing cognitively healthy individuals from those with PPA.

### 4.1 Classical features predictive of both FTLD‐4Rtau and AD

Classical features pertaining to fluency were found to impact classification performance within both PPA neuropathologies, aligning with previous findings showing that FTLD‐4Rtau pathology is typically characterized by slower, less fluent speech output.^25^, ^27^, ^51^, ^52^ Likewise, individuals with PPA due to AD are known to have reduced fluency compared to cognitively healthy participants.^16^, ^25^ Despite occurring in both PPA due to AD and FTLD‐4Rtau, fluency disruptions in the two disease pathologies likely stem from distinct mechanisms and from damage to different components of the language network. In FTLD‐4Rtau, atrophy in the IFG, supplementary motor area (SMA), and premotor regions disrupts articulatory planning, thereby affecting fluency.^53^, ^54^ Fluency disruptions in AD likely result from difficulties in word retrieval and phonological processing, which are associated with atrophy of the left posterior superior temporal gyrus (pSTG) and temporoparietal (TPJ) region.^55^, ^56^.

Lexical diversity and coherence impacted classification f‐scores within both PPA groups. Prior evidence suggests these measures are helpful in differentiating clinical variants of PPA from cognitively healthy controls^21^, ^57^ and may be particularly relevant in characterizing the language profile of PPA due to AD.^39^, ^58^ Additionally, the presence of fillers and non‐adverbial discourse markers significantly contributed to classification accuracy across both PPA groups, although these features had been previously linked exclusively to syndromes associated with AD.^58^, ^59^ In AD, these features may reflect word‐finding difficulties, potentially stemming from atrophy of the left pSTG, which is implicated in retrieving phonological representations.^60^ In FTLD‐4Rtau, production of fillers may stem from impaired articulatory and/or sentence planning, while increased coherence may reflect syntactic impairment—resulting in the production of simple, fixed syntactic structures.

Classification f‐scores within both groups of PPA were also influenced by production of closed‐class words, specifically pronouns and particles, including negations. These results align with findings linking reduced production of pronouns to syndromes frequently associated with FTLD‐4Rtau,^14^, ^15^, ^61^ and with evidence that the processing of closed class words largely relies upon the left inferior and middle frontal gyri,^62^, ^63^ which often are the earliest areas of cortical atrophy in FTLD‐4Rtau. Noun‐possessive dependencies also influenced classification accuracy for both neuropathologic groups with PPA, in accord with reports of difficulty producing possessive adjectives in various forms of PPA.^64^

### 4.2 Classical features predictive of either FTLD‐4Rtau or AD

Some features were associated with changes in f‐score for FTLD‐4Rtau but not for AD. These included the production of out‐of‐vocabulary words, which is likely a reflection of phonological and/or articulatory errors resulting in non‐words. Additional features distinctive of FTLD‐4Rtau included dependencies between verbs and their arguments, the usage of subordinating conjunctions (e.g., after, since), and average dependency distance. The importance of argument structure and syntactic dependencies in FTLD‐4Rtau aligns with evidence that cortical atrophy in FTLD‐4Rtau typically begins in the left IFG, which has been associated with processing of argument structure and long‐distance syntactic dependencies, as well as with increasing syntactic complexity.^65^, ^66^, ^67^, ^68^

Only one feature (i.e., presence of determiner‐noun dependencies) was uniquely associated with classification performance in the AD group. This finding is consistent with evidence linking the computation of local syntactic dependencies (such as those between nouns and determiners) to the left anterior and/or posterior STG,^69^, ^70^ which are early targets of atrophy in PPA due to AD.^25^ In our dataset, production of open‐class words was not predictive of AD neuropathology, a result not supported by the study that leveraged spontaneous speech to predict amyloid beta positivity on amyloid PET imaging.^39^ This discrepancy may be attributed to our inclusion of two neuropathologic diagnosis groups for individuals with PPA (AD and FTLD‐4Rtau), along with an additional group of cognitively healthy individuals. Our more rigorous and diversified group stratification may have reduced the importance of some features in favor of others.

### 4.3 Implications of the present study on differential diagnosis and disease management

The application of NLP and ML frameworks to narrative speech bears significant clinical utility, in that—together with currently available biomarkers—such methods can improve the accuracy of early differential diagnosis of PPA by offering a complementary tool for the early prediction of the underlying neuropathology. Current biomarkers for in vivo detection of disease pathology are limited to AD, while those for in vivo detection of other disease pathologies in PPA are still under development and remain far from being incorporated into standard clinical practice. The ability to predict PPA neuropathology in living individuals based on narrative speech samples collected in the early stages of disease would provide researchers and clinicians with additional support for guiding disease management and for selecting tailored treatment options as they become available. These results serve as proof of concept that applying NLP and ML tools to narrative speech samples can aid the differential diagnosis of PPA, provide crucial insights into underlying neuropathologic disease, and offer a possible avenue for determining study eligibility should disease‐specific clinical trials become available.

### 4.4 Limitations

A key direction of future research will be to encompass a broader range of underlying disease pathologies and neurological conditions. The present study focused exclusively upon distinguishing among PPA groups defined by either AD or FTLD‐4Rtau neuropathologic diagnosis; however, incorporating data from additional neuropathologies such as TDP‐43 and FTLD‐3Rtau (i.e., Pick's disease) or non‐PPA aphasic narratives would enhance both the granularity and scope of the classification models while increasing clinical relevance.

Our current analysis relied exclusively upon transcribed speech, which omits the rich acoustic information inherent to spoken language. Acoustic features such as speech rate, prosody, articulation errors, and fluency disruptions are salient features in some forms of PPA and may offer additional discriminatory value. A multimodal approach integrating textual and acoustic features may yield more comprehensive models, reflecting the multifaceted nature of language impairment in neurodegenerative disease.

Our current sample also exhibited class imbalance, which can influence classification outcomes. Though the dataset was weighted toward AD, the models nonetheless demonstrated comparable accuracy across diagnostic categories. Additionally, validation using a larger and fully independent test set would be preferable to assess broader generalizability. While we inferred meaning from metrics derived via leave‐one‐out cross‐validation, the coexistence of high feature dimensionality together with a relatively small number of participants does increase the risk of producing unstable estimates. The results should therefore be interpreted cautiously until validated in larger, fully pathologically confirmed cohorts, and future research should prioritize the collection of larger and more diverse datasets across clinical and pathologic groups to enhance model proficiency and clinical applicability.

## 5 CONCLUSIONS

The current study applies a constellation of NLP and ML tools to transcribed narrative samples with the principal aim of distinguishing PPA groups defined by underlying disease neuropathology (AD vs. FTLD‐4Rtau). Permutation‐based feature analysis revealed detailed patterns in speech production, demonstrating how predictive modeling can map specific features of narrative speech to disease pathology in PPA. Critically, features pertaining to verb–argument structure and long‐distance dependencies best separated FTLD‐4Rtau from other groups, whereas features describing local syntactic dependencies best discriminated AD from other groups. Features reflecting fluency, lexical diversity, and production of closed class words were crucial to differentiating both PPA groups from cognitively healthy participants. Our results, though preliminary, underscore the promise for the development of refined artificial intelligence frameworks that can enhance early detection and diagnosis of PPA neuropathologies and help determine eligibility for future disease‐specific clinical trials.

## CONFLICT OF INTEREST STATEMENT

The authors declare no conflicts of interest.

## CONSENT STATEMENT

The authors have reviewed and approved the submission of this manuscript. Informed consent was obtained from all human participants prior to trial enrollment

## Supporting information



Supporting Information

Supporting Information

## Data Availability

Anonymized data can be made available by submitting a collaborative request at https://www.brain.northwestern.edu/scientists‐students/collaborative‐request.html.
